# Erlotinib plus bevacizumab in EGFR-amplified metastatic solid tumors: results from the KOSMOS I, II study of molecular profiling–guided therapy in advanced cancers

**DOI:** 10.1007/s10147-026-03080-5

**Published:** 2026-06-15

**Authors:** Jiwon Lee, Jongmin Sim, Yun-Gyoo Lee, Gyeong-Won Lee, Hyun Ae Jung, Kyu-Pyo Kim, Tae-Yong Kim, Hyewon Ryu, Min-Hee Ryu, Mi-Sun Ahn, Minsuk Kwon, Bhumsuk Keam, Jeong Mo Bae, Sheehyun Kim, Harim Koo, Sun Young Kim, Jee Hyun Kim, Soohyeon Lee

**Affiliations:** 1 Division of Medical Oncology and Hematology, Department of Internal Medicine, Korea University College of Medicine, Seoul, Republic of Korea; 2 Department of Pathology, Korea University Anam Hospital, Korea University College of Medicine, Seoul, Republic of Korea; 3 Department of Internal Medicine, Kangbuk Samsung Hospital, Sungkyunkwan University School of Medicine, Seoul, Korea; 4 Division of Hematology and Oncology, Institute of Medical Science, Department of Internal Medicine, Gyeongsang National University Hospital, Jinju, Korea; 5 Division of Hematology-Oncology, Department of Medicine, Samsung Medical Center, Sungkyunkwan University School of Medicine, Seoul, 06351 Republic of Korea; 6 Department of Oncology, Asan Medical Center, University of Ulsan College of Medicine, Seoul, Korea; 7 Department of Internal Medicine, Seoul National University College of Medicine, Seoul National University Hospital, 101, Daehak-ro, Jongno-gu, Seoul, 03080 Republic of Korea; 8 Division of Hematology and Oncology, Department of Internal Medicine, Chungnam National University Hospital, Chungnam National University College of Medicine, Daejeon, South Korea; 9 Department of Hematology-Oncology, Ajou University School of Medicine, Suwon, Korea; 10 Department of Internal Medicine, Seoul National University Hospital, Seoul National University College of Medicine, Seoul, South Korea; 11 Cancer Research Institute, Seoul National University College of Medicine, Seoul, South Korea; 12 Department of Pathology, Seoul National University Hospital, Seoul National University College of Medicine, Seoul, Republic of Korea; 13 Department of Medical Science Convergence, Graduate School of Medical Science, University of Ulsan, Ulsan, Korea; 14 Department of Internal Medicine, Department of Genomic Medicine, Seoul National University Bundang Hospital, Seoul National University College of Medicine, Seongnam, Republic of Korea

**Keywords:** EGFR amplification, Drug repurposing, Metastatic solid tumors, Next-generation sequencing (NGS), Erlotinib plus Bevacizumab

## Abstract

**Purpose:**

Epidermal growth factor receptor (*EGFR*) amplification remains a controversial therapeutic biomarker. We report outcomes of erlotinib plus bevacizumab (E + B) in patients with *EGFR*-amplified metastatic solid tumors.

**Methods:**

Patients with metastatic solid cancer who had progressed after standard therapies and were treated with E + B based on central molecular tumor board recommendations in the KOSMOS trial were included. Only those with an *EGFR* copy number ≥ 3 were selected. The primary endpoint was the clinical benefit rate (CBR), while secondary endpoints included overall response rate (ORR), progression-free survival (PFS), overall survival (OS), and safety.

**Results:**

Between February 2021 and April 2024, 25 patients were treated with E + B (median EGFR copy 8.9; range, 3–76). Six tumor types were included, with colorectal cancer being the most prevalent (N = 14), followed by glioblastoma (N = 5), and other types (N = 6). Overall, four partial responses (PR) and seven cases of stable disease (SD) lasting over 16 weeks were observed, resulting in a CBR of 44.0% and ORR of 16%. The median PFS was 3.7 months (95% confidence interval [CI]: 1.7–4.6), whereas the median OS was 7.9 months (95% CI: 6.5 to not estimable). Six patients experienced one or more grade 3 adverse events related to E + B, including hypertension and mucositis.

**Conclusion:**

The E + B combination showed modest anti-tumor activity in patients with heavily pretreated *EGFR*-amplified solid cancers. While NGS may help identify new applications for existing drugs, additional investigations are warranted to validate the efficacy and benefit of E + B in this population.

**Supplementary Information:**

The online version contains supplementary material available at 10.1007/s10147-026-03080-5.

## Introduction

In precision medicine, cancer therapy has adopted a histology–agnostic approach, in which treatment decisions are primarily driven by tumor genomics rather than tumor type [1]. Evidence shows that shared molecular abnormalities exist across tumors originating from different anatomical sites [2–5]. The U.S. Food and Drug Administration (FDA) has approved drugs targeting specific biomarkers, including microsatellite instability-high (MSI-H)/mismatch repair deficient (dMMR), tumor mutational burden-high (TMB-H), and neurotrophic tyrosine receptor kinase (*NTRK*) gene fusions. This approach enables treatments tailored to the genetic characteristics of a tumor, regardless of the primary cancer type, offering more effective and precise therapies. Additionally, ongoing basket and umbrella trials are evaluating multiple drugs for various biomarkers within a single multi-cohort protocol [6, 7].

The epidermal growth factor receptor (EGFR), also known as human epidermal growth factor receptor 1 (HER1) or ErbB1, is a key receptor tyrosine kinase within the ErbB family, which includes ErbB2 (HER2), ErbB3 (HER3), and ErbB4 (HER4) [8]. EGFR is activated by specific ligands, such as epidermal growth factor (EGF) and transforming growth factor-alpha (TGF-α), which initiate downstream signaling pathways. As a result, EGFR significantly contributes to various cellular processes, including proliferation, survival, and differentiation, across multiple cancer types [8, 9]. EGFR overexpression and alterations, such as mutations and amplifications, are commonly observed in multiple cancers, including brain, lung, head and neck, breast, and colorectal cancer (CRC) [10]. Given EGFR’s critical role in oncogenesis, extensive efforts have been made to develop targeted therapies. These include monoclonal antibodies (mAbs) that inhibit the receptor’s extracellular domain and small-molecule tyrosine kinase inhibitors (TKIs) designed to block its tyrosine kinase activity [11, 12].

Although the EGFR pathway has been extensively investigated, the role of *EGFR* amplification as a predictive biomarker has not been fully established. A previous study reported that *EGFR* amplification was observed in approximately 8.5% (2423/28584) of patients with malignancies via cell-free DNA (cfDNA) analysis, with varying frequencies across different cancer types [13]. Among the nine patients with *EGFR* amplification who received anti-EGFR-based therapy, five exhibited a clinical response. In another study, *EGFR* amplification was observed in 5% (19/363) of patients with gastroesophageal adenocarcinoma; among the seven patients who received anti-EGFR therapy, four responded [14]. These findings suggest that *EGFR* amplification may act as a predictive biomarker. To date, prospective clinical data specifically evaluating anti-EGFR therapy in patients selected based on EGFR amplification remain very limited across solid tumor types, highlighting the need for biomarker-enriched prospective studies.

Various combination strategies have been explored to optimize EGFR pathway targeting by integrating EGFR-targeted therapies with other signaling pathways [15, 16]. Recent studies have investigated pathways, such as vascular endothelial growth factor (VEGF), the ErbB family, mesenchymal–epithelial transition (MET) inhibitors, and the phosphoinositide 3-kinase (PI3K)/ protein kinase B (AKT)/mechanistic target of rapamycin (mTOR) axis to enhance therapeutic efficacy and overcome resistance. Evidence suggests that VEGF upregulation is associated with resistance to EGFR-targeted therapies [17]. In *EGFR*-positive non-small cell lung cancer (NSCLC), some studies have indicated that combining erlotinib with bevacizumab may prolong progression-free survival (PFS) than monotherapy. This indicates that dual inhibition of these pathways could be a potential therapeutic strategy [18]. Nevertheless, conflicting results remain, highlighting the need for further investigation to determine optimal combination strategies.

The Korean Precision Medicine Networking Group conducted the KOSMOS and KOSMOS-II trials, which are prospective, pragmatic multi-cohort studies conducted at 31 medical centers across Korea, evaluating antitumor activity of molecular guided treatment in advanced cancers outside of its approved indication [19]. This study reports data from the KOSMOS and KOSMOS-II trial, specifically on patients with *EGFR* amplification who received a combination of erlotinib plus bevacizumab (E + B), to explore its potential as a predictive biomarker and therapeutic target.

## Methods

### Study design

The KOSMOS and KOSMOS-II studies are nationwide, prospective, pragmatic, multi-cohort studies, initiated with the KOSMOS pilot study in February 2021 and subsequently continued as the KOSMOS-II study. They are designed to screen patients with metastatic solid tumors for actionable genetic alterations (GAs) based on local next-generation sequencing (NGS) testing and to recommend molecularly guided therapies (MGTs) through a virtual molecular tumor board (MTB) convened weekly or biweekly. Overall, 32 sites participated in both KOSMOS and KOSMOS-II. MGT options were categorized into three tiers. Tier 1 included the therapeutic use of investigational drugs outside their approved indications, targeting actionable alterations with specific agents, such as alectinib for *ALK* fusion, atezolizumab for TMB-H, erlotinib ± bevacizumab for *EGFR* alterations, trastuzumab + pertuzumab or trastuzumab emtansine (T-DM1) for *ERBB2* amplification or overexpression, vemurafenib for *BRAF* V600 mutations, E + B for *FH* inactivating mutations, entrectinib for *ROS1* fusion, and pralsetinib for *RET* fusion. Tier 2 included alternative options using drugs permitted for treatment outside the indications approved by the Health Insurance Review and Assessment Service (HIRA), radiotherapy, or palliative care. Tier 3 comprised clinical trials matched to GAs as recommended by the MTB. The detailed design and methodology of the KOSMOS studies have been described in previous reports [19, 20].

### Patients and data collection

Patients eligible for KOSMOS met the following criteria: histologically confirmed advanced/metastatic cancer; progression on at least one line of therapy with no standard treatment available; NGS reports from the Ministry of Food and Drug Safety (MFDS)-accredited laboratories or those compatible with MFDS standards; age ≥ 19 years; and a life expectancy of at least 12 weeks. To be included in Cohort C, which was treated with E ± B, patients were selected based on the recommendation of the MTB. Among these, only those with *EGFR* amplification, defined as a copy number ≥ 3, were included in this analysis. As no universally validated cut-off for EGFR amplification currently exists, the MTB recommended E + B based on clinical judgment for each patient who had exhausted standard treatment options; the CN ≥ 3 threshold was applied in this analysis to define the study population.

### Treatment and assessments

Patients received 150 mg of erlotinib orally once daily and 15 mg/kg of bevacizumab intravenously triweekly until disease progression, unacceptable toxicity, or withdrawal of consent. Each treatment cycle lasted 21 days. Dose modifications for erlotinib were made in 50 mg decrements (150 mg → 100 mg → 50 mg) based on toxicity severity per the approved prescribing information. No dose reductions are recommended for bevacizumab; however, bevacizumab was withheld or permanently discontinued for specific adverse events as described in Supplementary Table S1. Tumor response was assessed every 8 weeks using RECIST version 1.1, except for patients with glioblastoma, for whom the Response Assessment in Neuro-Oncology (RANO) criteria were applied in conjunction with neuro-oncology specialists. Continuation of treatment beyond disease progression was permitted at the investigator’s discretion. Safety evaluations were conducted for all patients who received at least one dose of treatment, with adverse events assessed using the National Cancer Institute’s Common Terminology Criteria for Adverse Events (CTCAE) version 4.03.

### Study endpoints and statistical analysis

The primary endpoint was the clinical benefit rate (CBR), defined as a complete response (CR) or partial response (PR) or stable disease (SD) lasting for at least 16 weeks (SD16 +), as determined using RECIST version 1.1. Secondary endpoints included progression free survival (PFS), overall survival (OS), overall response rate (ORR), 1-year survival rate, and toxicity, assessed according to the CTCAE version 4.0. PFS and OS were calculated using the Kaplan–Meier method, and AEs were categorized and reported based on their frequency and proportion using standardized terminology. Ternary plots were generated to visualize response-associated patterns of genomic alterations across PR, SD, and PD groups; detailed methodology is described in the Supplementary Methods.

### Ethics

This study was conducted in accordance with the Declaration of Helsinki and approved by the institutional review boards of all participating centers, including the Institutional Review Board of Korea University Anam Hospital (No. 2022AN0416) Written informed consent was obtained from all patients prior to enrollment.

## Results

### Patient characteristics

Twenty-five patients with *EGFR* amplification across eight clinical sites were enrolled between February 2021 and April 2024. The median patient age was 61 years (range, 37–77) (Table 1). Six tumor types were treated, the most common being CRC (14 patients), followed by glioblastoma (5 patients), esophageal cancer (2 patients), head and neck cancer (2 patients), thymic carcinoma (1 patient), and small intestine cancer (1 patient). Seven (28%) patients were female. Ten patients had received 3 or more prior systemic therapies, 7 (28%) were previously treated with anti-EGFR therapy, and 17 (68%) were previously treated with anti-VEGF therapy. NGS platforms used to identify genomic alterations are listed in Table 1.

**Table 1:** Baseline characteristics of the study population

Characteristics	All patients (N = 25)
Median age (range), y	61 (37–77)
**Sex, n(%)**	
Female	7 (28%)
Male	18 (72%)
**Cancer type (%)**	
Colorectal	14 (56%)
*KRAS/NRAS* mutation	3
*RAF* mutation	1
*RAS/RAF* wild type	10
MSI status	
MSI-H	0
MSI-L	0
MSS	14
Glioblastoma	5 (20%)
Esophageal	2 (8%)
Head and neck	2 (8%)
Thymus	1 (4%)
Small intestine	1 (4%)
**No of prior lines of treatment (%)**	
1 Line	2 (8%)
2 Line	13 (52%)
3 Line +	10 (40%)
**Previous treatment**	
Anti-EGFR	7 (28%)
Among CRC patients^+^	7/14 (50%)
Anti-VEGF	17 (68%)
**NGS platform**	
Illumina Nextseq	18 (72%)
Illumina Microseq	2 (8%)
Illumina Hiseq	2 (8%)
Ion torrent	1 (4%)
Others	2 (8%)

^+^Among 14 CRC patients, 7 (50%) had received prior anti-EGFR monoclonal antibody therapy, all of whom were RAS/RAF wild-type

### Treatment outcomes

**Fig. 1:**
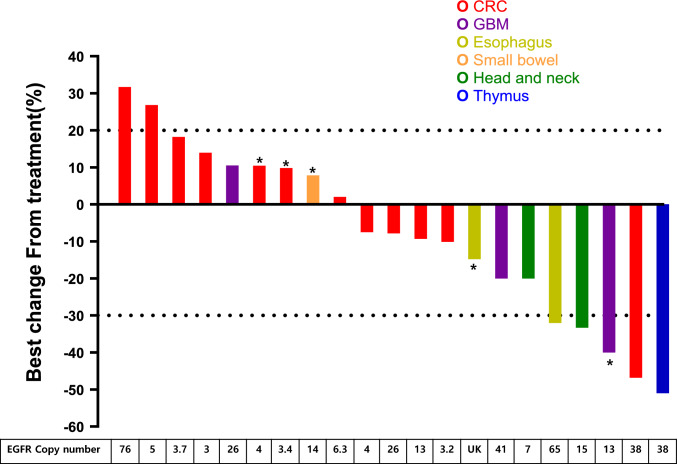
Waterfall plot showing the maximum percent change in tumor size from baseline among patients with measurable disease (N = 21).* Five patients with new lesion

In the entire population, the CBR was 44.0% (95% confidence interval [CI]: 24.4–65.1), and the ORR was 16% (95% CI: 6.4–34.7) (Table 2). Patients with CRC, who constituted the majority of the enrolled cases, had a CBR of 42.9%, with an ORR of 7.1%. In total, 4 patients achieved a PR, while 11 patients had SD, of whom 7 maintained SD for at least 16 weeks. In the four patients with PR, the tumor types included CRC, esophageal cancer, head and neck cancer, and thymic carcinoma. These patients showed high *EGFR* amplification, with *EGFR* copy numbers of 38, 65, 15, and 38 respectively. Fig 1 shows the maximum percent change in the target lesion size from baseline.

**Table 2:** Efficacy of erlotinib plus bevacizumab according to tumor types

	All patients	Colorectal cancer	Other types
**Patients enrolled (No.)**	25	14	11
**Best response, No. (%)**			
CR	0	0	0
PR	4	1	3
SD	11	7	4
SD16 +	7	5	2
PD	11	6	4
NA	0	0	0
Clinical Benefit Rate (CBR) (%)	44.0% (95% CI 24.4–65.1)	42.9% (95% CI 21.4–67.4)	45.5% (95% CI 21.3–72.0)
Objective response rate (ORR) (%)	16% (6.4–34.7)	7.1% (1.3–31.5)	27.3% (9.7–56.6)

**Fig. 2:**
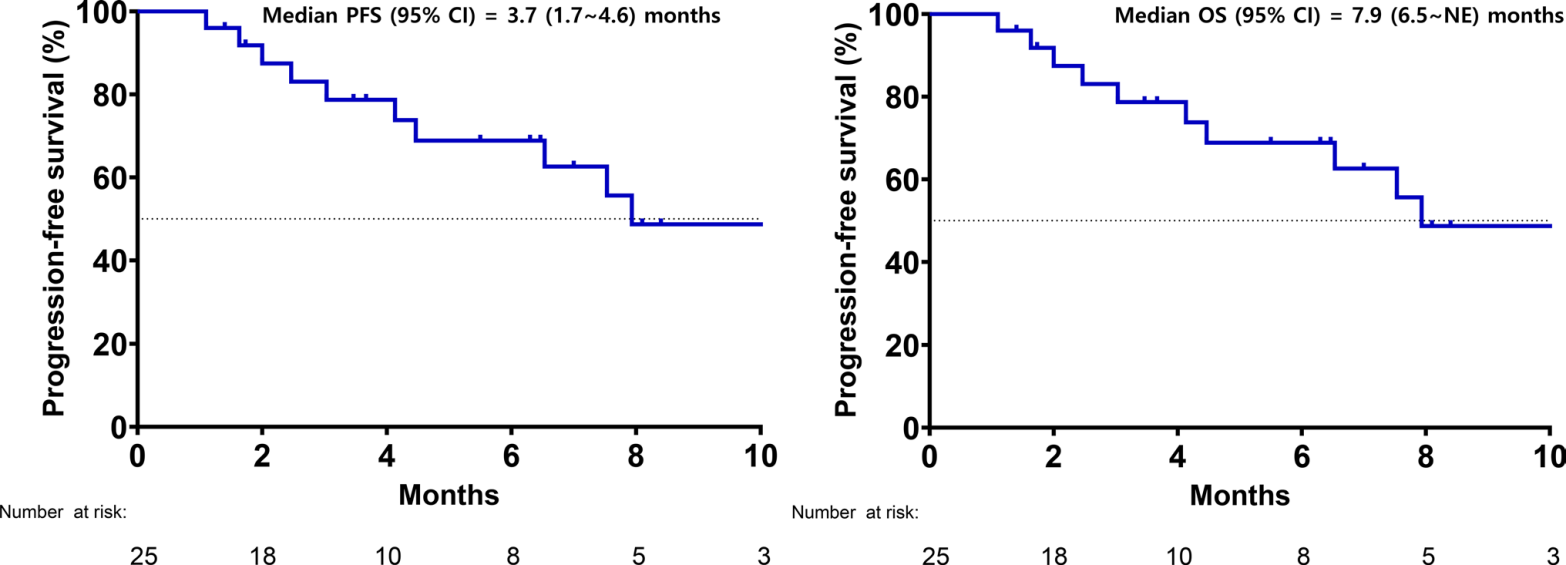
Median progression-free survival and Overall survival in 25 patients with EGFR amplification treated with Erlotinib + Bevacizumab

**Fig. 3:**
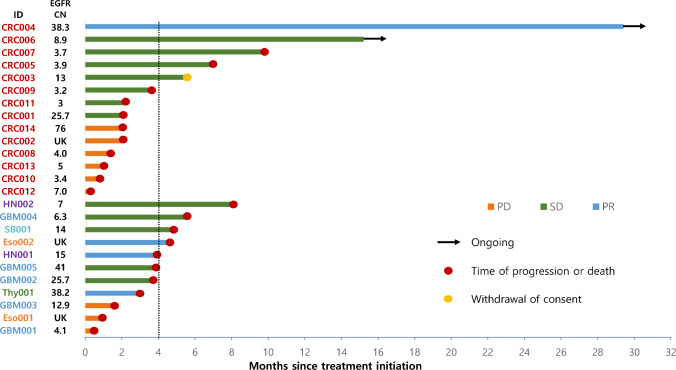
Swimmer’s plot of patients with EGFR amplification undergoing Erlotinib + Bevacizumab. The presence of ongoing response and dates of progressive disease are indicated

For all patients, the median PFS was 3.7 months (95% CI, 1.7–4.60) and the median OS was 7.9 months (95% CI, 6.5 to NE) (Fig. 2). Figure 3 shows treatment duration, progression time, and best response. Two CRC patients (CRC004 and CRC006) had maintained treatment for over one year by the data cut-off (May 2, 2024), both of whom were RAS/RAF wild-type.

### Safety

All the patients were included in the safety analysis (Table 3). Nine grade 3 AEs occurred in six patients; no grade 4 AEs were reported. Grade 3 AEs included diarrhea, mucositis, proteinuria, increased bilirubin levels, arthralgia, gastrointestinal (GI) bleeding, hypertension, AST/ALT elevation and fracture. The most common drug-related AEs were rashes (60%, 15/25), diarrhea (12%, 3/25), and mucositis (12%, 3/25).

**Table 3:** Treatment-related adverse events

Adverse events	All grades	%	Grade ≥ 3	%
Rash	15	60.0		
Diarrhea	3	12.0	1	4
Mucositis	3	12.0	1	4
Proteinuria	2	8.0	1	4
Arthralgia	2	8.0	1	4
GI bleeding	2	8.0	1	4
Fatigue	2	8.0		
Decreased appetite	2	8.0		
AST/ALT elevation	1	4.0	1	4
Increased bilirubin	1	4.0	1	4
Hypertension	1	4.0	1	4
Fracture	1	4.0	1	4
Dry skin	1	4.0		
Weight loss	1	4.0		
Nausea	1	4.0		

### Genomic profiling

The most predominant co-alteration in this cohort was *TP53* (15 of 24; 62.5%), followed by *APC, BRCA2, FLT3, ALK, ARID1A*, and *ATM* (Fig S1). A violin plot comparing *EGFR* copy numbers across patients with PR, SD, and progressive disease (PD) illustrated the distribution within each response group. Although no statistically significant differences were observed in the *EGFR* copy numbers among the three groups, a trend toward higher copy numbers was observed in patients with PR (Fig S2a). Patients with PD were characterized by the enrichment of *KIT, NOTCH1*, and *ERBB2* amplifications, and *KIT/MAP2K1* mutations, whereas patients with PR exhibited *FGFR3* and *MTOR* mutations (Fig S2b).

### Case series

#### CRC004 patient

A 59-year-old female diagnosed with synchronous sigmoid colon and low rectal cancer underwent surgical resection and adjuvant FOLFOX chemotherapy. Following a one-year disease-free interval, isolated liver metastasis was treated with resection and first-line FOLFIRI plus cetuximab. Further recurrences in the liver and lung were managed with additional surgeries and second-line FOLFOX plus bevacizumab. Subsequently, nodal metastases developed, for which third-line XELOX chemotherapy was administered, achieving partial response. However, disease progression eventually occurred. NGS analysis of surgical tissue revealed an EGFR copy number gain (copy number: 38). Based on this finding, she was enrolled in the KOSMOS trial and received E + B. A significant reduction in the size of the metastatic lymph node was observed, from 2.5 to 0.9 cm (Fig S3). She demonstrated sustained disease control for 18 months under E + B therapy, after which she underwent laparoscopic lymph node dissection. As of the data cut-off, she remains in a no-evidence-of-disease (NED) state.

### Thy001 patient

A 61-year-old male was diagnosed with thymic squamous cell carcinoma with supraclavicular and T3 vertebral metastases. The tumor was PD-L1 negative and showed EGFR amplification with a copy number of 38. He received first-line paclitaxel and carboplatin, achieving a partial response; however, disease progression occurred within two months, with new metastases to the liver, lungs, bones, lymph nodes, and maxilla. Second-line lenvatinib was initiated but failed to control progression. He was subsequently enrolled in the KOSMOS-2 trial and treated with E + B. A liver lesion decreased from 62 to 28 mm on follow-up imaging, indicating a radiologic partial response (Fig S4). Despite this, progressive disease developed in the lungs and bones, with a progression-free survival (PFS) of 3 months.

## Discussion

This study investigated the antitumor activity of E + B in patients with solid cancers who had failed standard therapy and had no appropriate treatment options. Of the 25 patients, 4 achieved PR, whereas 7 had SD16 + , which resulted in a CBR of 44.0% in patients with *EGFR* amplification. The median PFS and OS were 3.7 months and 7.9 months, respectively. In the safety analysis, grade 3 AEs were observed in 24.0% of the patients, with no grade 4 AEs reported. These side effects were consistent with previous studies and product documentation [21].

Several retrospective studies have reported the efficacy of anti-EGFR treatments in patients with GI cancer and *EGFR* amplification. In a multicenter retrospective study, 60 patients diagnosed with gastroesophageal adenocarcinoma and *EGFR* amplification received anti-EGFR therapy. Of these, 50 patients received mAbs, 8 patients received EGFR TKI, and 2 patients received both mAbs and EGFR TKI. This cohort yielded a 43% ORR and a median PFS of 4.6 months [22]. In another study that investigated the clinical response to anti-EGFR treatment in patients with CRC, eight of nine patients who showed an objective response to EGFR mAbs had *EGFR* amplification. In contrast, only one out of 21 non-responders showed *EGFR* amplification [23]. In a meta-analysis investigating the *EGFR* gene copy number as a potential predictive biomarker in patients with CRC, *EGFR* copy number gain was associated with improved outcomes of anti-EGFR mAbs in patients with wild-type *KRAS* [24]. Unlike these prior studies, which primarily investigated EGFR mAbs with or without chemotherapy, our population received an EGFR TKI combined with bevacizumab. Given that VEGF activation is a known mechanism of resistance to anti-EGFR therapy [25], dual inhibition of both pathways may provide synergistic effects, as supported by preclinical models demonstrating significant suppression of CRC tumor growth with combined anti-VEGF and anti-EGFR therapy [26].

Treatment efficacy varied considerably according to cancer type, EGFR copy number, and co-occurring genomic alterations. Among the four patients who achieved PR, three had squamous cell carcinoma histology (esophageal, head and neck, and thymic carcinoma) and one had CRC (CRC004). The single CRC responder was notable for an exceptionally high EGFR copy number (CN 38), RAS/RAF wild-type status, and ultimately achieved a complete radiologic response (NED). Consistent with this observation, partial responders across all tumor types had notably high EGFR copy numbers (range, 15–65), and a trend toward higher copy numbers among responders was observed (Figure S2a). These findings are consistent with prior reports of a positive association between EGFR copy number and response to EGFR TKIs in squamous cell carcinoma, including gefitinib in esophageal cancer [27] and afatinib in EGFR-amplified head and neck cancer [28], and support the hypothesis that high-level amplification may confer greater oncogene dependence.

In contrast, patients with progressive disease tended to harbor co-occurring alterations in ERBB2, NOTCH1, and KIT, as illustrated in the ternary diagram (Figure S2b). Among these, ERBB2 alterations have the most established mechanistic rationale as a driver of primary resistance to EGFR-directed therapy. ERBB2 amplification has been shown to confer resistance to EGFR-directed therapy through bypass activation of the HER2 signaling axis, and was enriched in KRAS/NRAS/BRAF/PIK3CA wild-type CRC tumors that failed to respond to cetuximab in patient-derived xenograft models [29, 30]. Activation of NOTCH signaling following EGFR inhibition has also been reported in preclinical models and in patients with acquired resistance to EGFR TKIs [31], suggesting a potential role as an alternative survival pathway. The clinical significance of KIT alterations in the context of EGFR-directed therapy resistance remains less established. Taken together, these co-occurring alterations may reflect activation of alternative or bypass signaling pathways contributing to primary resistance in a subset of patients, though the small sample size and tumor heterogeneity of the present study preclude definitive conclusions.

In the CRC subgroup specifically, the CBR was 42.9% and the ORR was 7.1%, despite heavily pretreated status including prior exposure to anti-EGFR and anti-VEGF therapies. Notably, following progression on standard irinotecan- and oxaliplatin-based chemotherapy, patients with CRC have limited treatment options, with trifluridine/tipiracil and regorafenib yielding poor response rates of only 2% and 1%, respectively [32, 33]. Against this backdrop, E + B may represent a promising option for a biomarker-selected subset of heavily pretreated EGFR-amplified CRC patients, particularly those with high copy numbers, RAS/RAF wild-type status.

In this study, patients with tumor types other than CRC exhibited a CBR of 45.5%, although the sample size was small and heterogeneous. In our cohort, only one of five patients with GBM achieved SD16 + , consistent with previously reported poor responses to EGFR-targeted therapy in this population, underscoring the need for novel biomarkers and therapeutic strategies [34, 35]. Prior studies of single-agent EGFR TKIs in recurrent GBM, including erlotinib [36] and gefitinib [37], have similarly demonstrated limited efficacy regardless of EGFR amplification status. These findings collectively suggest that EGFR-directed therapy has minimal activity in GBM, potentially due to insufficient blood–brain barrier penetration of erlotinib, with CSF/plasma ratios of approximately 5–7% reported across studies [38, 39], as well as activation of alternative downstream signaling pathways independent of EGFR inhibition.

This study had several limitations. First, as no universally validated cut-off for EGFR amplification currently exists, some patients with lower copy numbers (e.g., three) were included based on MTB clinical judgment. The optimal cut-off for defining amplification and predicting clinical benefit remains uncertain, which may have influenced treatment outcomes. Second, local NGS testing was employed across diverse platforms and was often performed at variable time points during each patient’s clinical course, rather than immediately prior to the initiation of E + B. This may have limited the real-time relevance of the genomic data. However, this approach reflects the diversity encountered in real-world clinical practice. Finally, the small sample size and heterogeneity in tumor types and prior treatments limit the generalizability of our findings. Future prospective studies should enrich for patients with squamous cell carcinoma histology and high EGFR copy numbers, where the signal for benefit appears strongest, and should incorporate standardized prospective genomic profiling to enable more rigorous biomarker analyses.

In conclusion, E + B demonstrated modest overall activity in unselected EGFR-amplified solid tumors (ORR 16%, median PFS 3.7 months). Durable responses were observed predominantly in patients with squamous cell carcinoma histology and high EGFR copy numbers, whereas co-occurring genomic alterations may have contributed to primary resistance in others. These findings support further prospective evaluation of E + B in biomarker-enriched cohorts with squamous histology and high-level EGFR amplification.

## Supplementary Information

Below is the link to the electronic supplementary material.

Supplementary file1 (DOCX 5441 kb)

## Data Availability

De-identified data that support the findings of this study are available from the corresponding authors upon reasonable request. Requests will be considered in accordance with institutional policies and participant consent.
