# Supplementary material for: Erlotinib plus bevacizumab in EGFR-amplified metastatic solid tumors: results from the KOSMOS I, II study of molecular profiling–guided therapy in advanced cancers
Source: Int J Clin Oncol. 2026 Jun 15;31(8):1668–77. doi: 10.1007/s10147-026-03080-5 (PMC13401561; doi:10.1007/s10147-026-03080-5)
Supplement: Supplementary file 1 — Supplementary file1 (DOCX 5441 kb) [file 10147_2026_3080_MOESM1_ESM.docx]

**Supplementary Fig 1.** Oncoplot for oncogenic somatic mutations.

**Supplementary Fig 2.** (a). Violin Plot of EGFR Copy Numbers Across Response Groups (PR, SD, PD)

(b) Ternary Diagram Showing Mutation Proportions Across Response Groups (PR, SD, PD)

**Supplementary Fig 3.** Contrast-enhanced CT images showing response of metastatic lymph node in the CRC004 case. (a) Baseline image showing a 2.5 cm metastatic lymph node. (b) Follow-up image after targeted therapy demonstrating marked decrease in size to 0.9 cm (blue arrow). The same imaging protocol was used in both scans.

**Supplementary Fig 4.** Contrast-enhanced CT images showing response of a metastatic liver lesion in the Thy001 case. (a) Baseline image showing a 6.2 cm lesion in the liver. (b) Follow-up image after targeted therapy demonstrating marked decrease in size to 2.8 cm (blue arrow). The same imaging protocol was used in both scans.

**Supplementary Methods**

**Response-Associated Genomic Alteration Patterns in Ternary Plots**

To assess response-associated patterns of genomic alterations, ternary plots were generated based on the relative frequencies of each alteration event across the three clinical response groups: partial response (PR), stable disease (SD), and progressive disease (PD). For each event, the number of patients harboring the alteration in each response group was calculated and normalized to the total number of patients with that event, yielding three proportions (f_PR,f_SD,f_PD) that satisfied the following equation:

f_PR+f_SD+f_PD=1.0"  (or 100%)"

These proportions were mapped onto ternary coordinates using barycentric transformation, where proximity to a given vertex indicated relative enrichment of the alteration in the corresponding response group. The size of each point was scaled according to the maximum absolute patient count observed among PR, SD, and PD for that event. Alterations were annotated by event class, with copy number variations (CNVs; gain/loss) and mutations (SNV/INDEL) distinguished by color and symbol, and the accompanying legend summarized alteration class, response-group axis labels, and point-size scale. All analyses and visualizations were performed in R (version 4.4.0) using the ggtern package.

**Supplementary table 1.**

| **Toxicity / Situation** | **Action** | **Notes** |
| --- | --- | --- |
| **Erlotinib (150 mg/day orally)** | | |
| Severe rash unresponsive to medical management | Withhold; resume at 50 mg dose reduction after resolution to ≤ Grade 1 | Dose reduction steps: 150 mg → 100 mg → 50 mg |
| Persistent severe diarrhea unresponsive to loperamide | Withhold; resume at 50 mg dose reduction after resolution to ≤ Grade 1 | Manage diarrhea with loperamide first |
| Grade 3–4 keratitis or persistent Grade 2 keratitis (>2 weeks) | Withhold; resume at reduced dose after resolution | Discontinue if corneal perforation occurs |
| Hepatic toxicity: total bilirubin >3× ULN or transaminases >5× ULN (no pre-existing hepatic impairment) | Withhold; consider discontinuation | For pre-existing hepatic impairment: bilirubin >2× ULN or transaminases >3× ULN |
| Severe renal toxicity (Grade 3–4) | Withhold; consider discontinuation | Monitor renal function periodically |
| Interstitial lung disease (ILD) | Permanently discontinue | Interrupt pending diagnostic evaluation for any new pulmonary symptoms |
| Gastrointestinal perforation | Permanently discontinue | — |
| Severe or life-threatening bullous/exfoliative skin toxicity | Permanently discontinue | — |
| **Bevacizumab (15 mg/kg IV every 3 weeks)** | | |
| Severe hypertension uncontrolled with medical management | Withhold until controlled; resume when medically managed | No dose reductions recommended per prescribing information |
| Nephrotic-range proteinuria (urine protein ≥3.5 g/24h) | Withhold; discontinue if proteinuria persists beyond treatment delay | Monitor urine protein prior to each dose |
| Gastrointestinal perforation | Permanently discontinue | — |
| Arterial thromboembolic event | Permanently discontinue | — |
| Hypertensive crisis or hypertensive encephalopathy | Permanently discontinue | — |
| Posterior reversible encephalopathy syndrome (PRES) | Permanently discontinue | — |
| Wound healing complications / surgical procedures | Withhold at least 28 days prior to elective surgery; discontinue if wound healing complications develop | — |
| Severe hemorrhage | Permanently discontinue | — |

Abbreviations: ULN, upper limit of normal; ILD, interstitial lung disease; PRES, posterior reversible encephalopathy syndrome.
